# VEGFR-1 Overexpression Identifies a Small Subgroup of Aggressive Prostate Cancers in Patients Treated by Prostatectomy

**DOI:** 10.3390/ijms16048591

**Published:** 2015-04-16

**Authors:** Maria Christina Tsourlakis, Puya Khosrawi, Philipp Weigand, Martina Kluth, Claudia Hube-Magg, Sarah Minner, Christina Koop, Markus Graefen, Hans Heinzer, Corinna Wittmer, Guido Sauter, Till Krech, Waldemar Wilczak, Hartwig Huland, Ronald Simon, Thorsten Schlomm, Stefan Steurer

**Affiliations:** 1 Institute of Pathology, University Medical Center Hamburg-Eppendorf, Martinistrasse 52, D-210246 Hamburg, Germany; E-Mails: m.tsourlakis@uke.de (M.C.T.); puya.khosrawi@hotmail.de (P.K.); philipp.weigand@uke.de (P.W.); m.kluth@uke.de (M.K.); c.hube@uke.de (C.H.-M.); s.minner@uke.de (S.M.); c.koop@uke.de (C.K.); c.wittmer@uke.de (C.W.); g.sauter@uke.de (G.S.); t.krech@uke.de (T.K.); w.wilczak.de (W.W.); s.steurer@uke.de (S.S.); 2 Martini-Clinic, Prostate Cancer Center, University Medical Center Hamburg-Eppendorf, Martinistrasse 52, D-210246 Hamburg, Germany; E-Mails: graefen@uke.de (M.G.); heinzer@uke (H.He.); hhuland@uke.de (H.Hu.); tschlomm@uke.de (T.S.); 3 Department of Urology, Section for translational Prostate Cancer Center, University Medical Center Hamburg-Eppendorf, Martinistrasse 52, D-210246 Hamburg, Germany

**Keywords:** VEGFR-1, TMPRSS2:ERG, PTEN, TMA, prostate cancer

## Abstract

The VEGFR-1 is suggested to promote tumor progression. In the current study we analyzed prevalence and prognostic impact of the VEGFR-1 by immunohistochemistry on a tissue microarray containing more than 3000 prostate cancer specimens. Results were compared to tumor phenotype, ETS-related gene (ERG) status, and biochemical recurrence. Membranous VEGFR-1 expression was detectable in 32.6% of 2669 interpretable cancers and considered strong in 1.7%, moderate in 6.7% and weak in 24.2% of cases. Strong VEGFR-1 expression was associated with *TMPRSS2:ERG* fusion status as determined by fluorescence *in situ* hybridization (FISH) and immunohistochemistry (*p* < 0.0001 each). Elevated VEGFR-1 expression was linked to high Gleason grade and advanced pT stage in *TMPRSS2:ERG* negative cancers (*p* = 0.0008 and *p* = 0.001), while these associations were absent in *TMPRSS2:ERG* positive cancers. VEGFR-1 expression was also linked to phosphatase and tensin homolog (*PTEN*) deletions. A comparison with prostate specific antigen (PSA) recurrence revealed that the 1.7% of prostate cancers with the highest VEGFR-1 levels had a strikingly unfavorable prognosis. This could be seen in all cancers, in the subsets of *TMPRSS2:ERG* positive or negative, *PTEN* deleted or undeleted carcinomas (*p* < 0.0001 each). High level VEGFR-1 expression is infrequent in prostate cancer, but identifies a subgroup of aggressive cancers, which may be candidates for anti-VEGFR-1 targeted therapy.

## 1. Introduction

Prostate cancer is the most prevalent cancer in men in Western societies [1]. Although the majority of prostate cancers behave in an indolent manner, a small subset is highly aggressive and requires extensive treatment [2,3]. Established pre-therapeutic prognostic parameters are limited to Gleason grade and tumor extent on biopsies, preoperative prostate specific antigen (PSA), and clinical stage. Because these data are statistically powerful, but often insufficient for optimal individual treatment decisions, it is hoped that a better understanding of disease biology will eventually lead to the identification of clinically applicable molecular markers that enable a more reliable prediction of prostate cancer aggressiveness. At the same time, there is a need for alternative anticancer drugs targeting specific structures of prostate cancer cells.

Vascular endothelial growth factor receptor 1 (VEGFR-1) is a candidate for both a prognostic biomarker and representing a suitable target molecule for specific cancer therapy [4]. The VEGF family is composed of five structurally related factors (VEGF-A, -B, -C, -D and phosphatidylinositol-glycan biosynthesis class F (PIGF)), which act as the primary activators of angiogenesis by binding to tyrosine kinase receptors VEGF-receptor 1, 2 and 3 (VEGFR1–3). Whereas the roles of VEGFR-2 and VEGFR-3 as direct stimulators of angiogenesis (VEGFR-2) and lymphangiogenesis (VEGFR-3) have been characterized thoroughly, the function of VEGFR-1 is less clear. The VEGFR-1 gene encodes two different proteins: membrane-bound FGFR1 (Flt-1) and a soluble form termed sVEGFR-1. Both forms have been shown to negatively regulate VEGFR-2 through high-affinity binding of VEGFs, which consequently become unavailable for VEGFR-2 (reviewed in [5]). However, it has been suggested that VEGFR-1 might indirectly promote tumor cell growth by activation of monocytes and macrophages, which invade the tumor and produce VEGFs and cytokines, leading to angiogenesis and lymphangiogenesis via activation of VEGFR-2 and VEGFR-3 [6,7].

VEGFR-1 is described to be expressed on tumor cells in many tumor types, including lymphoma [8], leukemia [9], multiple myeloma [10], melanoma [11], non-small cell lung cancer [12], colon cancer [13], pancreatic cancer [14] and breast cancer [4]. In several cancer entities, such as renal cell cancer [15], squamous cell cancer [16,17], non-small cell lung cancer [18], colon cancer [19] and breast cancer [20], VEGFR-1 was shown to be linked to disease outcome. In prostate, differential expression between cancerous and normal epithelium was described [21,22,23,24]. In addition, studies on 113, 40, and 79 cancers had suggested a possible link between high VEGFR-1 expression levels and unfavorable tumor phenotype and poor disease outcome [21,22,25]. However, this observation was not confirmed by others analyzing cancers from 15 patients [23].

To further clarify the clinical relevance of VEGFR-1 expression in prostate cancer by immunohistochemistry (IHC), we took advantage of our preexisting tissue microarray (TMA) containing >3000 prostate cancer specimens connected to a database with extensive clinical follow up. Our findings demonstrate that high levels of VEGFR-1 protein expression are strongly linked to an adverse phenotype and early PSA recurrence of prostate cancer.

## 2. Results

### 2.1. Technical Issues

A total of 2669 (82%) tumor samples were interpretable in our TMA analysis. Reason for non-informative cases (592 spots, 18%) included lack of tissue samples or absence of unequivocal cancer tissue in the TMA spot.

### 2.2. VEGFR-1 Expression in Prostate Cancer

**Figure 1:**
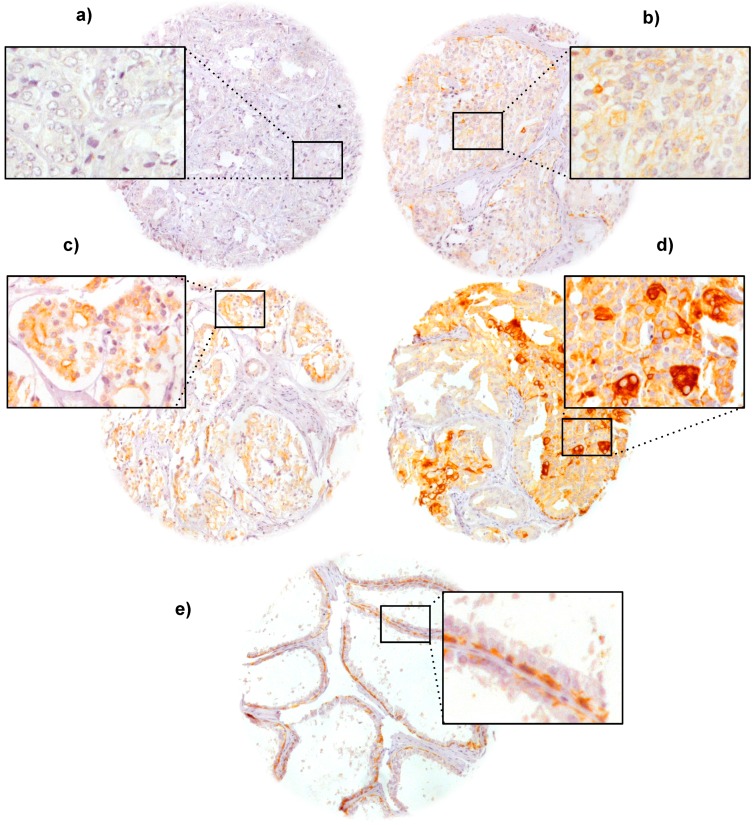
(**a**) Negative; (**b**) weak; (**c**) moderate; and (**d**) strong VEGFR-1 immunostaining in prostate cancer as well as (**e**) in a normal prostate. Magnification 70× and 240× for the insets.

VEGFR-1 immunostaining was localized in the membrane and the cytoplasm. Positive VEGFR-1 staining was also regularly recorded in basal cells of non-neoplastic prostate epithelium and in basal cells of prostatic intraepithelial neoplasia (PIN). Positive VEGFR-1 staining was seen in 869 of our 2669 (32.6%) interpretable prostate cancers and was considered weak in 24.2%, moderate in 6.7% and strong in 1.7% of cancers, while 1800 (67.4%) did not show any membranous VEGFR-1 reactivity. Representative images of positive and negative VEGFR-1 immunostainings are given in Figure 1.

### 2.3. Association with TMPRSS2:ERG Fusion Status and ERG Protein Expression

**Figure 2:**
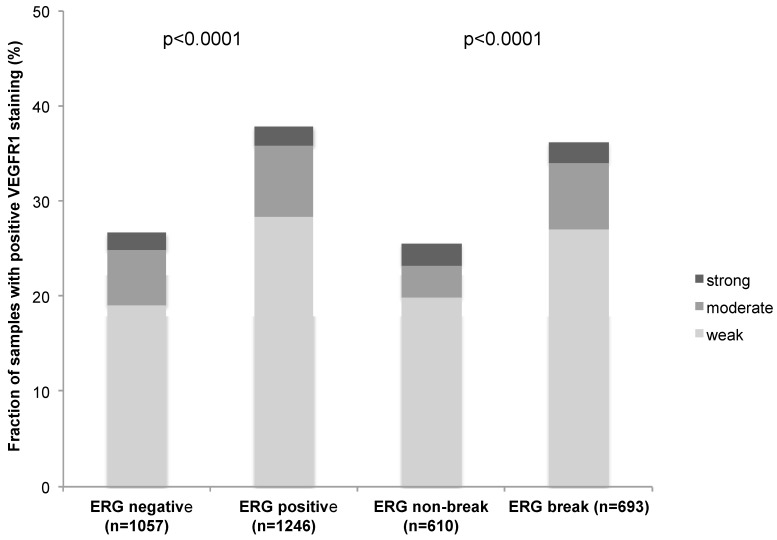
Correlation between VEGFR-1 expression and ERG-fusion probed with IHC and FISH.

To evaluate whether VEGFR-1 expression is associated with ERG status in prostate cancers, we used data from previous studies [26]. Data on *TMPRSS2:ERG* fusion status obtained by fluorescence *in-situ* hybridization were available from 1303 patients and by immunohistochemistry from 2303 patients. VEGFR-1 expression was significantly linked to *TMPRSS2:ERG* rearrangement and ERG expression (*p* < 0.0001 each, Figure 2).

### 2.4. Association with Tumor Phenotype

Increased VEGFR-1 expression was significantly linked to high Gleason grade (*p* = 0.03) and advanced pathological tumor stage (*p* < 0.0001), if all tumors were jointly analyzed (Table 1).

**Table 1:** Association between VEGFR-1 expression and cancer phenotype.

Parameter	Evaluable (N)	Immunostaining (%)				*p* Value
		Negative	Weak	Moderate	Strong	
All cancers	2669	67	24	6.7	1.7	
Tumor stage						
pT2	1728	69	23	7.0	1.0	<0.0001
pT3a	553	68	23	7.0	2.0	
pT3b	326	63	29	4.6	3.7	
pT4	34	50	29	5.9	15	
Gleason grade						
≤3 + 3	1156	69	23	7.3	1.1	0.03
3 + 4	1165	66	26	6.8	1.7	
4 + 3	273	69	23	4.8	3.7	
≥4 + 4	47	68	23	2.1	6.4	
Lymph node metastasis						
N0	1316	67	26	5.3	1.6	0.05
N+	84	57	32	4.8	6.0	
PSA preoperative						
<4	401	63	27	8.0	2.2	0.23
4–10	1435	67	24	7.3	1.3	
11–20	564	69	23	5.7	2.5	
>20	204	72	22	4.4	1.5	

Subgroup analysis of ERG positive and ERG negative cancers revealed, that these associations were largely retained in the subsets of ERG negative and positive cancers (Supplementary Tables S1 and S2).

### 2.5. Association with Other Key Genomic Deletions

**Figure 3:**
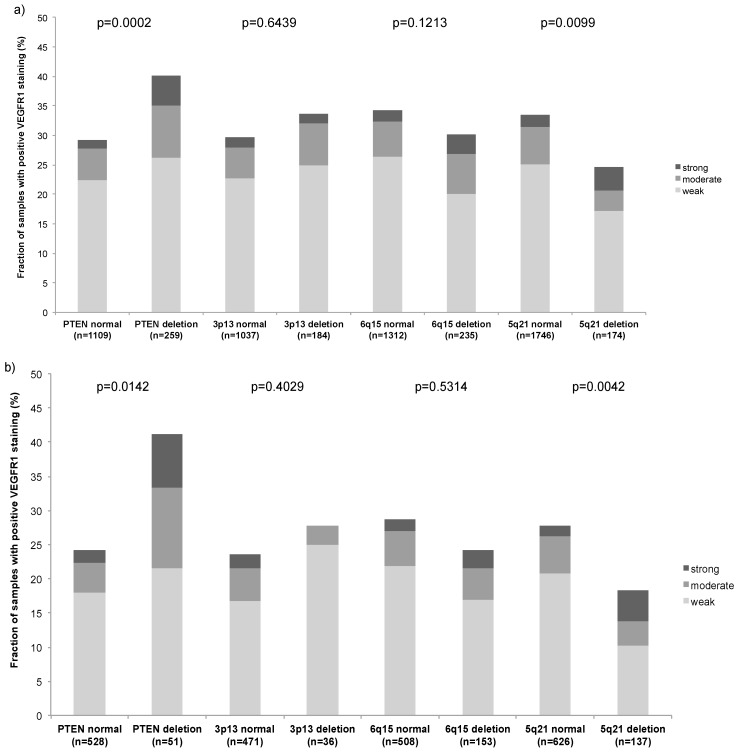

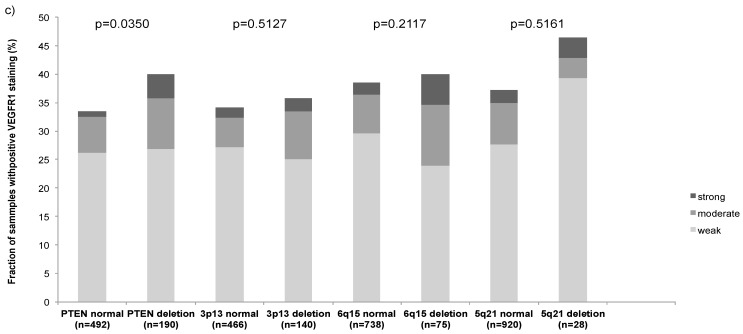
VEGFR-1 expression by IHC *versus* PTEN, FOXP1, MAP3K7 and CHD1 deletions by FISH in (**a**) All cancers; (**b**) ERG negative; and (**c**) ERG positive.

Earlier studies had provided evidence for distinct molecular subgroups of prostate cancers defined by *TMPRSS2:ERG* fusions and several genomic deletions. We as well as others described a strong link of *PTEN* and 3p13 deletion to ERG positivity and of 5q21 and 6q15 deletions to ERG negativity [27,28,29,30]. To study, whether VEGFR-1 data expression might be particularly linked to one of these genomic deletions, VEGFR-1 data were compared to preexisting findings on *PTEN* (10q23), *FOXP1* (3p13), *MAP3K7* (6q15) and *CHD1* (5q21) deletions. High VEGFR-1 staining was linked to *PTEN* deletions in the analysis of all tumors (*p* = 0.0002; Figure 3a), but also in the subgroup of ERG negative (*p* = 0.0142; Figure 3b) and ERG positive cancers (*p* = 0.0350; Figure 3c).

### 2.6. Association with PSA Recurrence

**Figure 4:**
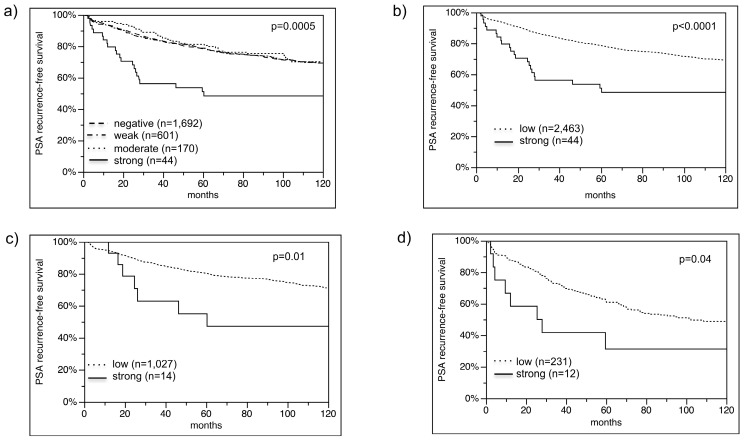
(**a**) Biochemical recurrence is associated with strong VEGFR-1 staining; (**b**) negative, weak and moderate grouped in low; (**c**) PTEN normal subset; and (**d**) PTEN deleted subset.

Follow-up data were available for 2507 patients with interpretable VEGFR-1 immunostaining on the TMA. There was a statistically significant association between high VEGFR-1 expression and early PSA recurrence if all tumors were analyzed (*p* = 0.0005; Figure 4a). The analysis revealed that unfavorable disease outcome was seen in particular in cancers with very high VEGFR-1 expression levels. Because of their similar clinical behavior, cancers with negative, weak and moderate VEGFR-1 expression, were subsequently combined as “low” for further analyses (Figure 4b). Separate analyses of molecularly defined cancer subgroups such as *PTEN* deleted and non-deleted prostate cancers revealed, that high level VEGFR-1 expression remained to be strongly associated with early PSA recurrence in these subsets (Figure 4c,d). Multivariate analysis revealed that strong VEGFR-1 expression remained a significant predictor of disease outcome (*p* = 0.02), if pathological tumor stage (*p* < 0.0001), Gleason grade (*p* < 0.0001), nodal stage (*p* < 0.0001), resection margin (*p* < 0.0001) and pre-operative PSA (*p* = 0.05) were included into the analysis (Table 2).

## 3. Discussion

In this study, IHC was applied to a TMA to evaluate VEGFR-1 expression in prostate cancer and normal prostatic epithelium. The TMA approach is optimal for IHC studies, because TMAs enable maximal experimental standardization. In this study more than 3000 prostate cancer specimens were analyzed in one day in one experiment using one set of reagents at identical concentrations, temperatures and exposure times. Moreover, all TMA sections were cut within hours on a single day immediately before staining to avoid unequal tissue reactivity to antibody binding [31,32]. Finally one pathologist interpreted all IHCs on one day to optimally standardize staining interpretation.

**Table 2:** Multivariate analysis of established prognostic parameters and VEGFR-1 expression status.

Parameter	RR	95% CI	*p* Value
Tumor stage			
pT3a *vs.* pT2	1.9	1.5–2.4	<0.0001
pT3b *vs.* pT3a	1.8	1.4–2.2	
pT4 *vs.* pT3b	1.4	0.9–2.1	
Gleason grade			
3 + 4 *vs.* ≤3 + 3	2.4	1.8–3.3	<0.0001
4 + 3 *vs.* 3 + 4	2.3	1.8–2.8	
≥4 + 4 *vs.* 4 + 3	1.4	1.0–2.1	
Nodal stage			
pN1 *vs.* pN0	1.9	1.5–2.5	< 0.0001
Resection margin status			
R1 *vs.* R0	1.5	1.2–1.8	<0.0001
Pre-operative PSA (ng/mL)			
4–10 *vs.* <4	1.1	0.8–1.6	0.05
11–20 *vs.* 4–10	1.1	0.9–1.4	
>20 *vs.* 11–20	1.2	0.9–1.5	
VEGFR-1			
strong *vs.* low	1.9	1.1–3.0	0.02

Our analysis revealed that VEGFR-1 was consistently and strongly expressed in basal cells of normal prostate epithelium and PIN. Secretory epithelial cells were negative. In cancer cells, VEGFR-1 expression was found in 32.6% of 2669 interpretable prostate cancers, indicating that VEGFR-1 can be activated in a fraction of secretory cells as a consequence of malignant transformation. The frequency of detectable VEGFR-1 staining is substantially lower in our study than in four earlier publications reporting VEGFR-1 expression in 97.5%, 100% and 100% analyzing 40, 15 and 113 prostate cancers and in 100% of 16 pelvic lymph node metastasis [21,22,23,25]. It is most likely, that these differences are due to the use of different antibodies and staining procedures. For our study, we selected the rabbit polyclonal antibody ab2350 raised against the *C*-terminal polypeptide not present in the soluble form of the VEGFR-1 protein, which does not react with the phosphorylated form of the protein. In contrast, Mao *et al.* [22] and Woolard *et al.* [24] used antibodies with a presumable broader binding spectrum, raised against the recombinant VEGFR-1 Ser27-His687 in goats. That varying antibody conditions lead to significant changes in the rate of positive cases was previously shown. For example the frequency of positive p53 immunostaining varied between 4% and 61% based on the experimental protocol [33]. A high variation of immunostaining is all the more expected if antibodies are compared that bind to different epitopes of the target protein.

The analysis of our large tumor cohort revealed that high VEGFR-1 expression was significantly linked to advanced tumor stage and high Gleason grade. However, the difference between the groups was rather small. For example, moderate to strong VEGFR-1 expression was detectable in 8% of pT2, 9% of pT3a and 8% of pT3b cancers. However, the majority of published investigations found associations with Gleason grade [21] and/or pT stage [21,22,25], while some investigations failed to reveal associations with pT stage [23] and/or Gleason grade [22,23].

Only 1.7% of 2669 interpretable cancers were considered to have a strong VEGFR-1 expression in this study. Only these highest expression levels were strongly linked to unfavorable prognosis, while cancers with low or moderate VEGFR-1 expression did behave as cancers without detectable VEGFR-1 expression. It is obvious that studies analyzing substantial smaller patient cohorts would not be able to identify such a small fraction of cancers as a relevant subgroup. Accordingly, two earlier studies failed to find associations between VEGFR-1 expression and disease outcome [21,22]. However, one study on 79 patients reported a prognostic impact of VEGFR-1 expression in pelvic lymph node negative prostate cancer [25]. The striking prognostic impact of high VEGFR-1 expression is consistent with studies suggesting an oncogenic role of this protein. For example, VEGFR-1 was found to promote migration of tumor cells through an Src-dependent pathway linked to activation of focal adhesion [34]. VEGFR-1 is also hypothesized to enable the development of cancers metastases by activation and pre-metastatic localization in distant organs of bone marrow-derived hematopoietic progenitor cells expressing VEGFR-1 (reviewed in [35]). Moreover, several studies suggest that VEGFR-1 might indirectly promote tumor cell growth by activation of monocytes and macrophages, which invade the tumor and produce VEGFs and cytokines, leading to angiogenesis and lymphangiogenesis via activation of VEGFR-2 and VEGFR-3 [6,7].

The mechanisms for VEGFR-1 overexpression applying to prostate cancer are not completely understood. The strong association particularly between low-level (weak to moderate) VEGFR-1 expression and ERG positivity suggests that overexpression of the ERG transcription factor might contribute to VEGFR-1 activation in prostate epithelial cells. In fact, a strong interaction with ERG was earlier demonstrated for VEGFR-2. In a study on transgenic Xenopus embryos and COS-7 cells, it was shown that ERG together with KLF2 (a member of the Krüppel-like factor transcription regulator family) synergistically activated transcription of VEGFR-2 [36]. *PTEN* deletions and other alterations of the PI3K/AKT signaling pathway may also contribute to high level VEGFR-1 expression in prostate cancer. Both PTEN and VEGFR-1 negatively regulate PI3K/AKT signaling activity in a feedback control loop [37], with intact PTEN being an inhibitor of VEGFR1 expression [38]. This explains the strong link between high level VEGFR-1 expression and *PTEN* deletions in our cancers. That high level VEGFR-1 expression is associated with poor disease outcome both in *PTEN* deleted and in *PTEN* undeleted cancers is further suggesting that a more complex dysregulation of PI3K/AKT signaling by multiple independent alterations of pathway components may have a clinically relevant additive impact.

The striking success of new targeted drugs raise the hope that prostate cancer patients might eventually also benefit from such an approach [39]. However, the few clinical trials in prostate cancer using drugs targeting Epidermal Growth Factor Receptor (EGFR) [40,41], VEGFR-2 [42] and HER2 [43,44,45] were discouraging so far. VEGFR-1 is another potential target molecule for specific cancer therapy [4]. IMC-18F1 (icrucumab), a human monoclonal antibody against VEGFR-1 tested in a Phase I study is described to have antitumor activity [46]. Our data suggest that at least 1.7% of prostate cancer patients might be excellent candidates for benefiting from such a therapy, once the drug should prove to be effective. Although a fraction of 1.7% may appear low, prostate cancer may become a relevant application of such new drugs given its high prevalence.

## 4. Experimental Section

### 4.1. Patients

Radical prostatectomy specimens were available from 3261 patients, undergoing surgery between 1992 and 2005 at the Department of Urology and the Martini Clinics at the University Medical Center Hamburg–Eppendorf (Table 3). Analysis of patient and corresponding histopathological data for research purposes, as well as construction of tissue microarrays from archived diagnostic left-over tissues, was approved by local laws (HmbKHG, §12,1) and by the local ethics committee (Ethics commission Ärztekammer Hamburg, WF-049/09 and PV3652). According to local laws, informed consent was not required for this study. Patient records/information was anonymized and de-identified prior to analysis. All work was carried out in compliance with the Helsinki Declaration. Follow-up data were available for a total of 3058, ranging from 1 to 228 months (mean 72.1). Prostate specific antigen values were measured following surgery and PSA recurrence was defined as the time point when postoperative PSA was at least 0.2 ng/mL and increasing at subsequent measurements. All prostate specimens were analyzed according to a standard procedure, including a complete embedding of the entire prostate for histological analysis [33]. The TMA manufacturing process was described before [47]. In short, one 0.6 mm core was taken from a representative tissue block from each patient. The tissues were distributed among 7 TMA blocks, each containing 144 to 522 tumor samples. For internal controls, each TMA block also contained various control tissues, including normal prostate. The molecular database attached to this TMA contained results on ERG expression in 2539, ERG break apart FISH analysis in 1474 [26] and deletion status of CHD1 [30] in 2197, MAP3K7 (6q15) in 1754 [27], PTEN (10q23) in 1447 [29] and FOXP1 (3p13) in 1290 [48] cancers.

**Table 3:** Clinico-pathological features of arrayed prostate cancers.

Parameter	*n* = 3261 on TMA	*n* = 2891 with Clinical Follow-up
Follow-up (months)		
Mean	72.1	
Median	68.9	
Range	1–219	
Age (years)		
<50	83	78
50–60	998	912
60–70	1807	1699
>70	175	169
Pretreatment PSA (ng/mL)		
<4	513	478
4–10	1673	1544
11–20	641	608
>20	225	212
pT category (AJCC 2002)		
pT2	2080	1907
pT3a	609	579
pT3b	372	361
pT4	42	42
Gleason grade		
≤3 + 3	1426	1307
3 + 4	1311	1238
4 + 3	313	297
≤4 + 4	55	49
pN category		
pN0	1544	1492
pN+	96	93
pNx	1457	1298
Surgical margin		
Negative	2475	2295
Positive	627	594

### 4.2. Immunochemistry

Freshly cut TMA sections were immunostained on one day and in one experiment. Primary antibody specific for VEGFR-1 (polyclonal rabbit ab2350, Abcam, Cambridge, UK; dilution 1:450) was applied, slides were deparaffinized and exposed to heat-induced antigen retrieval for 5 min in an autoclave at 121 °C in pH 7.8. Tris-EDTA-Citrate buffer. Bound antibody was then visualized using the EnVision Kit (Dako, Glostrup, Denmark). The staining intensity (0, 1+, 2+, 3+) and the fraction of positive tumor cells were recorded for each tissue spot. A final score was built from the two variables as previously described [26,49]: negative scores had staining intensity of 0, weak ones had a staining intensity of 1+ in ≤70% of tumor cells or 2+ in ≤30% of tumor cells; moderate had staining intensity of 1+ in >70% of tumor cells, staining intensity of 2+ in >30% and ≤70% of tumor cells or staining intensity of 3+ in ≤30% of tumor cells and strong scores had staining intensity of 2+ in >70% of tumor cells or staining intensity of 3+ in >30% of tumor cells.

### 4.3. Statistics

Statistical calculations were performed using JMP9 statistical software (SAS Institute, Inc., Cary, NC, USA). Contingency tables were calculated with the chi2-test to search for associations between molecular parameters and tumor phenotype. Survival curves were calculated according to Kaplan–Meier. The Log-Rank test was applied to detect significant survival differences between groups. Cox proportional hazards regression analysis was performed to test the statistical independence and significance between pathological, molecular and clinical variables.

## 5. Conclusions

Our study shows that strong VEGFR-1 expression identifies a small subgroup of highly aggressive prostate cancers. Given its role as a possible drug target, VEGFR-1 has strong potential both as a prognostic and therapeutic target in prostate cancer.

## Supplementary Materials

Supplementary materials can be found at http://www.mdpi.com/1422-0067/16/04/8591/s1.
