# Supplementary material for: VEGFR-1 Overexpression Identifies a Small Subgroup of Aggressive Prostate Cancers in Patients Treated by Prostatectomy
Source: Int J Mol Sci. 2015 Apr 16;16(4):8591–606. doi: 10.3390/ijms16048591 (PMC4425098; doi:10.3390/ijms16048591)
Supplement: Supplementary file 1 [file ijms-16-08591-s001.pdf]

Supplementary Information

Table S1. Association between VEGFR-1 expression and phenotype in the ERG negative subset.

Parameter	Evaluable (N)	Immunostaining (%)				*p* Value
		Negative	Weak	Moderate	Strong	
All cancers	1057	73	19	5.9	1.8	
Tumor stage						
pT2	701	76	18	5.6	0.9	0.0008
pT3a	200	75	15	7.5	2.5	
pT3b	128	63	28	4.7	4.7	
pT4	16	44	31	13	13	
Gleason grade						
≤3 + 3	474	77	17	5.1	0.4	0.001
3 + 4	420	69	21	7.6	2.1	
4 + 3	123	75	16	4.9	4.1	
≥4 + 4	28	61	29	0	11	
Lymph node metastasis						
N0	517	73	22	4.1	1.6	0.03
N+	34	53	29	8.8	8.8	
PSA preoperative						
<4	142	75	18	4.9	2.8	0.87
4–10	541	73	19	6.7	1.5	
>10	255	75	18	5.5	2.4	
>20	95	69	24	5.3	1.1	

Table S2. Association between VEGFR-1 expression and phenotype in the ERG positive subset.

Parameter	Evaluable (N)	Immunostaining (%)				*p* Value
		Negative	Weak	Moderate	Strong	
All cancers	1246	62	28	7.5	2.0	
Tumor stage						
pT2	755	63	28	8.2	1.6	0.09
pT3a	303	62	29	7.3	1.7	
pT3b	159	61	31	5.0	3.1	
pT4	15	53	27	0	20	
Gleason grade						
≤3 + 3	497	62	28	8.5	2.0	0.41
3 + 4	604	62	29	7.5	1.7	
4 + 3	116	62	29	4.3	4.3	
≥4 + 4	15	80	20	0	0	
Lymph node metastasis						
N0	655	63	29	6.6	1.8	0.27
N+	40	55	38	2.5	5.0	
PSA preoperative						
<4	190	56	33	9.0	2.6	0.11
4–10	684	62	28	8.5	1.5	
>10	245	62	29	5.7	2.9	
>20	93	74	20	3.2	2.2	
